# Ablation Compared to Pharmacological Treatment for the Reduction of Atrial Fibrillation Recurrence: A Meta-Analysis

**DOI:** 10.7759/cureus.62728

**Published:** 2024-06-19

**Authors:** Alan D Kaye, Nicholas T Jones, Tyler Tran, Munira E Khaled, Sean Tilmon, Michael Lieu, Joseph Drinkard, Yair Lopez Torres, Edwin Herron, Shahab Ahmadzadeh, Sahar Shekoohi, Giustino Varrassi

**Affiliations:** 1 Department of Anesthesiology, Louisiana State University Health Sciences Center, Shreveport, USA; 2 Department of Medicine, Louisiana State University Health Sciences Center, Shreveport, USA; 3 Department of Pain Medicine, Paolo Procacci Foundation, Rome, ITA

**Keywords:** af, arrhythmias, medication, ablation, atrial fibrillation

## Abstract

Atrial fibrillation (AF) is one of the most common heart arrhythmias, and due to its variable presentation, detecting and treating AF appropriately can reduce some of its serious complications. Among treatment options, surgical ablation and antiarrhythmic drug therapy are two of the most widely used choices. A systematic review and meta-analysis were conducted to examine the rates of AF recurrence in those treated with ablation compared to pharmacological treatment. Google Scholar and PubMed were searched for study trials published within the last decade that calculated the recurrence of AF symptoms in patient groups that received ablation or pharmacological treatment. Selected studies were analyzed in RevMan 5.4 software (The Cochrane Collaboration, London, England, UK), and each study's odds ratio and overall odds ratio were calculated using a 95% confidence interval. A total of seven studies with 2324 patients were analyzed for the meta-analysis, with 1162 patients receiving ablation and 1162 patients receiving pharmacological treatment. There was a statistically significant decrease in the recurrence of AF in the ablation group compared to the pharmacological treatment group, with a pooled odds ratio of 0.24 (CI 95% 0.14-0.39). AF treated with ablation was more effective in reducing AF recurrence than general pharmacological treatment.

## Introduction and background

Atrial fibrillation (AF) represents aberrations in the electrical currents of the heart, giving rise to arrhythmias with diverse symptoms and serious complications. AF is characterized by unregulated rapid contractions of the heart's atrial chambers and dysregulated ventricular contractions [1]. The mechanism of onset is hypothesized to be small reentrant propagations of action potentials, and the two currently supported mechanisms for the pathology of reentry into AF are centered around reentry rotors or multiple independent shallow waves, but further discussion is beyond the scope of this review [2]. Risk factors for the development of AF include those that are non-modifiable, such as family history, age, and male sex, and those that are modifiable, including alcohol use, a sedentary lifestyle, hypertension, smoking, and obesity [2,3]. When AF presents, patients most commonly experience a feeling of chest palpitations, typically after a triggering event or while at rest. However, AF may also be asymptomatic, allowing it to manifest in severe complications due to the risk of untreated AF [4]. AF is associated with an increased risk of death, dementia, heart failure, and stroke [5,6]. One study estimated that the risk of stroke or a transient ischemic attack in patients with AF is increased fivefold [7]. Thrombogenesis involves the stagnation of blood, the left atrial appendage, and inflammation, leading to endothelial dysfunction, which increases the risk of thrombus formation in the left atrium. After thrombus formation, the high-pressure systemic system and the heart's left chambers can easily dislodge the thrombus, transforming it into an embolus that can precipitate a stroke [2,7].

AF not only affects millions of patients worldwide, but it also has a significant financial impact. According to Karnik et al., the healthcare-associated costs of AF rose to $26 billion in 2008 and are estimated to nearly triple to $70 billion in 2030. About 6.5 million Americans over the age of 20 are affected by this disease, with an estimated 1.5 million more by 2030. At the age of 55, the lifetime risk for American patients rises to one in every three patients, while African American patients have a lifetime risk of one in every five patients [8]. As the statistics above demonstrate, AF is the most common stable heart arrhythmia in the United States. This study serves to illuminate some of the current treatment options for AF while comparing benefits and risks. The modifiable and non-modifiable risk factors for AF are also considered when discussing a treatment plan. Treatment of AF commonly consists of a surgical option involving ablation and pharmacological treatment for rate and rhythm control [9,10]. Rhythm control pharmacotherapy aims to maintain normal sinus rhythm in patients with AF, with Class Ic and III antiarrhythmic drugs typically used as first-line treatment. Class Ic drugs include flecainide or propafenone and work by blocking cardiac sodium channels, decreasing the slope of the action potential, and thus reducing excitation. Class III drugs include sotalol, amiodarone, and dofetilide. These work by inhibiting potassium channels and prolonging the QT interval [11]. Rate control pharmacotherapy aims to keep a person’s heart rate below 110 beats per minute so the reduction in stroke volume that occurs in AF is not exacerbated by faster heart rates. First-line agents for rate control are beta blockers, while secondary treatments include non-dihydropyridine calcium channel blockers and cardiac glycosides such as digoxin [12].

Catheter ablation is a surgical intervention for AF, often used when refractory to antiarrhythmic drug therapy. In this procedure, radiofrequency lesions are directed toward the orifices of the pulmonary vein until it is electrically disconnected from the left atrium [13]. Success rates vary, but some studies have shown that 76% of patients were free of AF post-ablation compared to only 19% on drug therapy alone [14]. However, both antiarrhythmic drug therapy and surgical ablation are not without significant side effects. Many antiarrhythmic drugs carry the cardiovascular side effects of bradycardia, hypotension, and Torsades de Pointes. As a result, many are contraindicated in patients who have ischemic or structural heart disease, heart failure, or other arrhythmias such as Wolff-Parkinson-White. Furthermore, numerous antiarrhythmic drugs inhibit the CYP system, creating potential adverse reactions with other medications that may require routine hepatic and renal monitoring [15,16]. Multiple studies have reported the risk of major complications in ablation being anywhere from 2% to 5%, with the most notable complications including tamponade, pneumothorax, and pulmonary vein stenosis requiring further intervention [17,18]. Life-threatening complications were present in just under 1% of ablation-treated AF [19]. After four years, AF recurrence on antiarrhythmic drug therapy alone was found by one study to be about 64%, compared to 36.5% for catheter ablation [20].

This systematic review and meta-analysis, therefore, attempt to compare the recurrence of AF in patients with ablation when compared to antiarrhythmic drug therapy alone.

## Review

Methods

Study Design

For this study, the population chosen includes patients greater than 18 years old with symptomatic AF and at least one episode of AF detected on electrocardiography within the last 24 months. For our analysis, we define intervention as catheter ablation. We will compare this to the patients treated with pharmacological treatment. The outcome studied is the incidence of the recurrence of AF in both treatment groups.

Inclusion and Exclusion Criteria

For our chosen topic, we established the following inclusion criteria: (1) articles must be from the last 10 years and (2) reported total counts of treatment and control groups with outcomes. The exclusion criteria are as follows: (1) conference abstracts, reviews, and letters to the editor; (2) animal studies; (3) unpublished articles/theses and incomplete studies; (4) articles that reported results for a sample size of less than 10; and (5) articles where the entire patient population had characteristics that would impact the study (e.g., hospitalization).

Search Strategy

**Figure 1:**
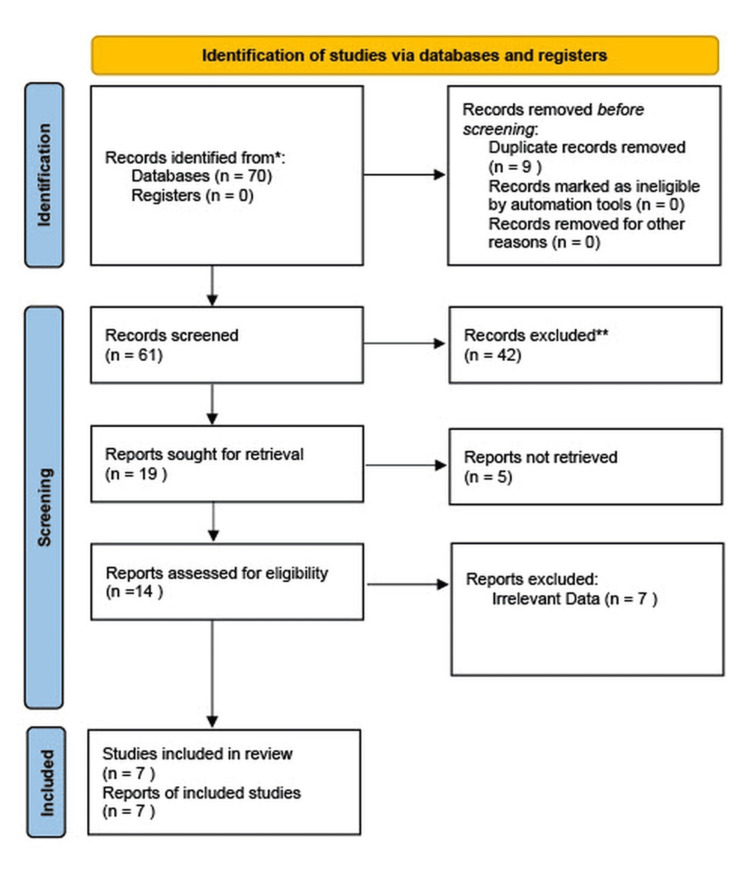
PRISMA diagram showing the study selection and inclusion PRISMA: Preferred Reporting Items for Systematic Reviews and Meta-Analysis

We performed a systematic search of PubMed and Google Scholar using the keywords AF, arrhythmias, medication, ablation, and AF for randomized controlled trials that were published between January 1, 2013, and August of 2023. The present meta-analysis adheres to the Preferred Reporting Items for Systematic Reviews and Meta-Analysis (PRISMA) guidelines. The studies were screened for duplicates, titles reviewed, and abstracts screened until a final full-text review was performed on the remaining articles to determine their use in the analysis (Figure 1).

Data Extraction and Quality Assessment

The studies were reviewed for the following information: (1) study characteristics; (2) ablation or pharmacological treatment being studied; and (3) analysis data (TP, FP, FN, and TN numbers). The qualities of the studies were assessed following Cochrane’s quality assessment tool [21]. Studies with high-risk evaluations for a particular signaling question in any domain were considered to have a high risk of bias for that domain. The five key domains were random sequence generation, allocation concealment, blinding of outcome assessment, incomplete outcome data, and selective reporting.

Statistical Analysis

The analysis was performed on the data from seven accepted studies. The event in question was defined as the recurrence of AF symptoms as recorded by individual studies. The control for each study is regarded as pharmacological treatment, and the experimental is ablation-based treatment techniques. The values for each criterion were recorded in 2x2 tables, and a meta-analysis was performed using RevMan 5.4 software (The Cochrane Collaboration, London, England, UK). The odds ratio was found for each study, as was the overall pooled odds ratio, each with a 95% confidence interval. The results were then displayed in a forest plot and a funnel plot.

Results

Search Results

From the two databases, 70 articles were identified for further evaluation (50 from PubMed and 20 from Google Scholar), of which nine were removed after reviewing for duplicate records. After reading the titles and abstracts, 42 articles were excluded for various reasons. Of the remaining 19 studies, 12 were excluded on the grounds of being irrelevant to our focused topic or not having full text available. The remaining studies qualified qualitative and quantitative synthesis. Finally, only seven studies (Table 1) met the inclusion criteria after screening the full text. The total population size for all studies combined is 2324 patients, with 1162 patients in the ablation group and 1162 patients in the control groups (seven studies).

**Table 1:** Study characteristics of the students included in the meta-analysis

Author (year)	Year	Sample size		Type of intervention and control	Location
		Treatment	Controls		
Andrade et al. [22]	2021	154	149	Cryothermy balloon ablation vs. pharmacological treatment (flecainide was most frequently prescribed, and 69.1% of patients were on monotherapy)	British Columbia, Canada
Jones et al. [23]	2013	26	26	Catheter ablation vs. rate control pharmacological treatment (beta-blocker and/or digoxin)	London, United Kingdom
Wazni et al. [24]	2021	99	104	Cryoballoon ablation vs. rhythm control therapy (Class I or III agents)	Cleveland, OH
Morillo et al. [25]	2014	61	66	Radiofrequency vs. antiarrhythmic therapy (69% received flecainide, 25% propafenone, and 16.4% tried more than 1)	Europe and North America
Packer et al. [26]	2021	400	378	Ablation vs. antiarrhythmic drug therapy (80% on rhythm control medications (propafenone 9%, flecainide 16%, sotalol 17%, dofetilide 13%, Amiodarone 44%, Dronedarone9%, Other 2%)	Rochester, MN
Wu et al. [27]	2021	321	327	Catheter ablation vs. pharmacological treatment (amiodarone or propafenone)	China
Di Biase et al. [28]	2016	101	102	Catheter ablation vs. amiodarone	New York, USA

Atrial fibrillation recurrence

**Figure 2:**
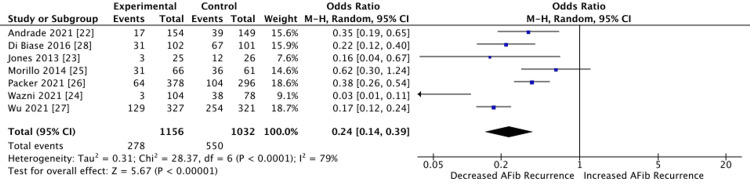
Individual and pooled odds ratio for all studies included in the meta-analysis for the reduction of AF with ablation procedures when compared to pharmacological management AF: atrial fibrillation

The analysis of the studies is reported in Figure 2. A statistically significant association was found in the reduction of AF recurrence when ablation is compared to normal pharmacological treatment, with a pooled odds ratio of 0.24 (CI 95% 0.14-0.39). Additionally, the analysis showed a relatively high heterogeneity between trials, with a calculated I^2^ of 79%.

Quality assessment

**Figure 3:**
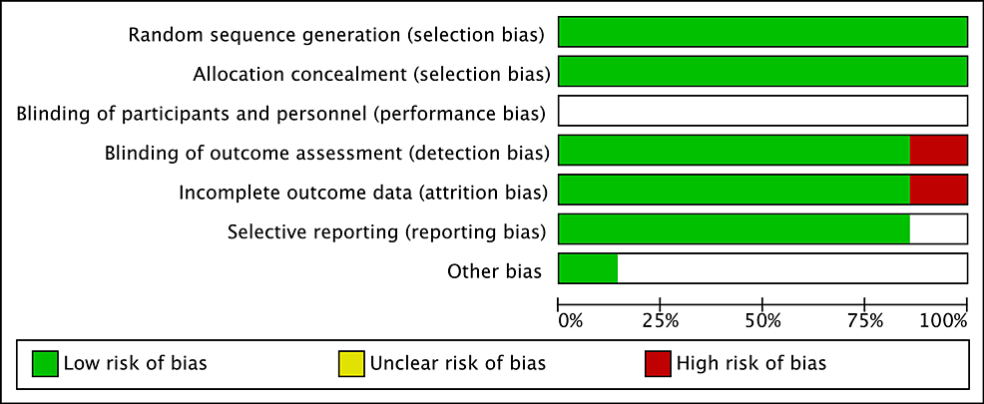
Quality assessment of the seven included articles for the treatment of AF via ablation or pharmacological treatment AF: atrial fibrillation

A detailed assessment of the quality of the seven included studies is included in Figure 3. The included studies showed a low risk of bias with random sequence generation, allocation concealment, and selective reporting. Blinding outcome assessment displayed high-risk bias in one of the seven studies cited. Incomplete outcome data showed high-risk bias in one of the seven studies cited. The blinding of participants and personal data displayed an unclear bias in all studies due to the intention to treat. All participants were informed of the treatment they were receiving after the randomization of the group and allocation concealment as the interventions began. Therefore, the overall study quality is acceptable for this meta-analysis.

Discussion

AF can be a fatal cardiac arrhythmia due to its sequelae if left untreated. This meta-analysis sought to determine which standard of care, pharmacological treatment or ablation, decreases the risk of recurrence to a significantly greater degree. Our findings demonstrate that ablation significantly decreases the risk of recurrence of the abnormal rhythm in patients with AF compared to pharmacological treatment. This meta-analysis further demonstrates that ablation is a more definitive treatment for AF.

There are some limitations to our study. First, the selection criteria for the included studies excluded confounding patient populations, which reduces the applicability of our meta-analysis to those patient populations. These excluded groups include patients with a past medical history of congestive heart failure, myocardial infections, or a previous ablation. Second, the compared pharmacological treatments were not standardized across all studies. Finally, the third limitation appears from the intent to treat protocol, where some patient groups received a second ablation related to inadequate control of AF.

Our results have reminded us to continue focusing on more definitive methods for the treatment of AF. Although ablation is significantly better at reducing recurrent AF than pharmacological treatment, neither treatment is completely curative of the condition due to the risk of recurrence in the ablation group. Additionally, further studies should investigate the risk of recurrence in the above-excluded patient populations, which were those with heart failure, myocardial infarctions, or previous ablations. Patients with a history of heart failure have an increased risk of AF, and ablations have already been proven to improve the quality of life in those groups; however, there is a paucity of literature showing a comparison between pharmacological treatment and ablation in this patient population [29].

## Conclusions

A total of seven studies with 2324 patients were analyzed for the meta-analysis, with 1162 patients receiving ablation and 1162 patients receiving pharmacological treatment. There was a statistically significant decrease in the recurrence of AF in the ablation group compared to the pharmacological treatment group, with a pooled odds ratio of 0.24 (CI 95% 0.14-0.39). AF treated with ablation was more effective in reducing AF recurrence than general pharmacological treatment. Overall, our meta-analysis shows that ablation for AF reduces the risk of recurrence significantly more than pharmacological management in the general population.
